# Bilateral Florid Papillomatosis of the Nipple: An Unusual Indicator for Metachronous Breast Cancer Development—A Case Report

**DOI:** 10.1155/2014/432609

**Published:** 2014-01-12

**Authors:** Walid Sasi, Dibyesh Banerjee, Kefah Mokbel, Anup K. Sharma

**Affiliations:** ^1^St. George's University of London, Cranmer Terrace, London SW17 0RE, UK; ^2^The London Breast Institute, The Princess Grace Hospital, London W1U 5NY, UK

## Abstract

Adenoma or florid papillomatosis of the nipple (FPN) is a rare benign disease which has histopathological features similar to those of a mammary papillary carcinoma. Here, we report a rare case of bilateral florid papillomatosis of the nipple and breast cancer, with a literature review.

## 1. Introduction

Adenoma or florid papillomatosis of the nipple (FPN) is a rare benign disease which clinically resembles Paget's disease of the nipple and has histopathological features similar to those of a mammary papillary carcinoma [1]. In this report, we discuss a rare case of bilateral florid papillomatosis of the nipple with unilateral breast cancer, followed by a brief literature review.

## 2. Case Report

A 63-year-old Caucasian woman presented to our breast clinic with a cracked right nipple and a chronic yellow discharge for 1 year. She had a left side mastectomy with Latissimus Dorsi flap reconstruction 2 years ago for breast cancer. Histopathological examination of the left mastectomy specimen reported features of left nipple florid papillomatosis along with multifocal ductal carcinoma *in situ* with apocrine features and microinvasive changes. Twelve years prior to that, she had a benign cyst removed from her left breast. Her mother died of ovarian cancer.

Findings on clinical examination were those of eczematous-like changes of her right nipple with crusting and nipple inversion. A yellow discharge could be expressed on examination and no palpable lumps were found in her right breast or either axillae. Her right breast mammogram showed heterogeneous glandular parenchyma which was unchanged compared to previous examinations. No new suspicious mammographic features were identified. The patient has subsequently undergone a major (total) duct excision with a specimen size of 30 × 27 × 17 mm.

**Figure 1:**
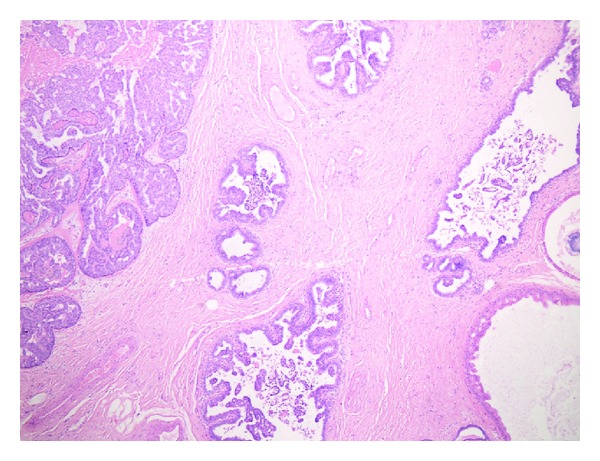
Papillomatosis, ×40.

**Figure 2:**
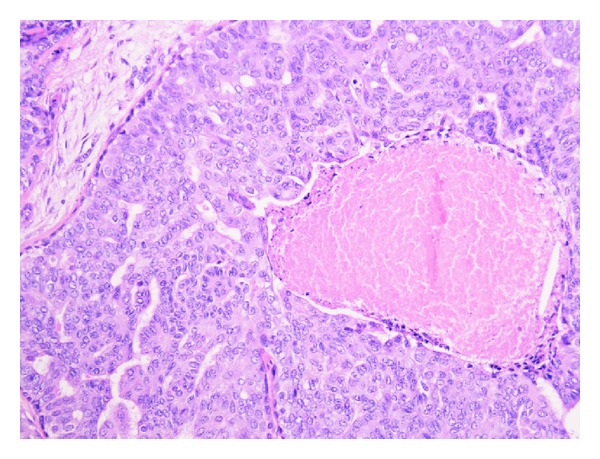
Area of necrosis, ×200.

**Figure 3:**
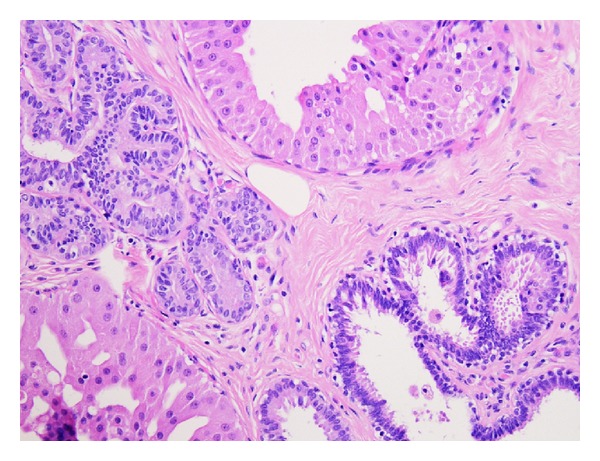
Areas of apocrine change, ×200.

**Figure 4:**
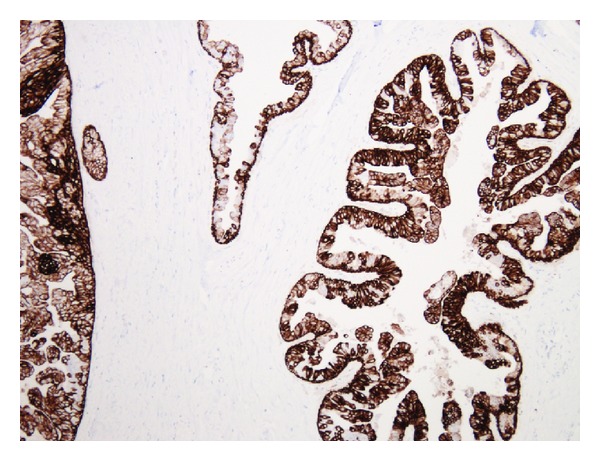
CK 5/6 immunostaining, ×200.

**Figure 5:**
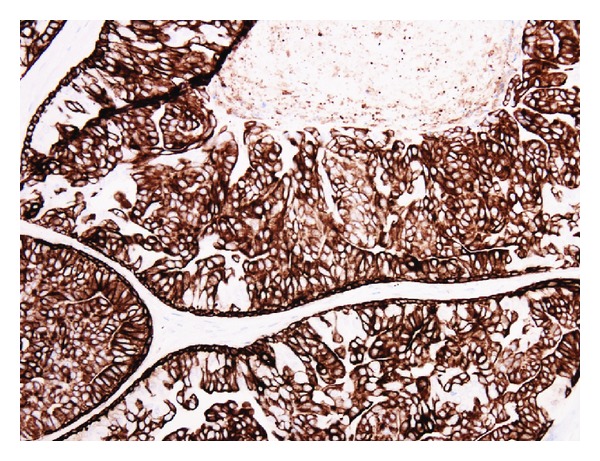
CK 5/6 immunostaining, ×200.

Histology of the breast tissue included major nipple ducts in the breast tissue, several of which showed florid epithelial hyperplasia with papillary hyperplasia in some areas (Figure 1). Ducts expanded and occluded by solid sheets of cells with focal necrosis were also seen, with periductal fibrosis (Figure 2). Apocrine changes were focally seen (Figure 3). In the first 3 sequential slices, there appeared to be a fairly well-defined nodule suggesting a major duct (or nipple) adenoma. Immunostaining showed strong membranous staining with CK 5/6, indicating preservation of basal/myoepithelial layer (Figures 4 and 5). The features were those of a subareolar sclerosing duct papillomatosis (florid papillomatosis of the nipple). The presence of solid areas with central necrosis and similar findings in the other breast two years ago with concurrent high grade DCIS were distinctly unusual features; however, expert opinion concurred with our diagnosis of duct papillomatosis.

This patient's case was discussed at our multidisciplinary meeting and it was decided that close followup is the best option to detect any future changes in her right breast.

## 3. Discussion

The term “papillomatous breast lesions” refers to benign proliferative epithelial breast lesions with underlying papillary architecture. The papillary layers are composed of glandular epithelial cells, basal myoepithelial layers, and fibrovascular cores. Basement membranes enclose these layers in a “papillary pattern.” Such lesions can either be central (located in major nipple/subareolar ducts or large segmental ducts) or peripheral (located in dilated ductal and lobular units). In a significant proportion of mammary papillomatous lesions, atypical changes can occur which constitute atypical proliferative tissue of the ductal type (to be distinguished from the papillary type of ductal carcinoma *in situ*).

About 17% of all mammary papillomatous lesions are associated with synchronous intraductal or invasive cancer [2]. However, it is also important to note that such lesions may also indicate a subsequent metachronous cancer. As a result, some authors have classified papillomatous mammary lesions on minimally invasive biopsy as “B3” to indicate this important association and the need for full excision [2].

In a case series analysed by Rosen and Caicco [3], 14% of patients with FPN have developed breast carcinoma in the same breast, and out of these, 29% had invasive carcinomas which appeared to arise from FPN lesions and 57% had concurrent invasive carcinomas separate from FNP lesions, while a single patient (14%) has developed diffuse ipsilateral intraductal carcinoma 10 years after FPN excision. Ono et al. have reported a case of subareolar ductal carcinoma that coexisted with FPN [4].

Our patient has a unique presentation with respect to her bilateral disease and the previously excised left-sided multifocal intraductal carcinoma. Although there were no atypical histopathological features or uniform loss of basal cell markers (CK 5/6), revision of previous literature series indicated the presence of a risk for developing malignant changes in up to 14%–17% of patients with FPN, even in the absence of cellular atypia at the time of FPN biopsy. Furthermore, there is also the low risk of developing subsequent carcinoma following excision of FPN which warrants clinical followup.

## 4. Conclusion

To the best of our knowledge, this is the first reported case of bilateral florid papillomatosis of the nipple (FPN) with an associated metachronous cancer. FPN is a rare benign disease entity which is proved to be associated with the risk of development of intraductal and invasive mammary carcinomas, either at the time of diagnosis or even years following the benign disease excision. Disease excision (in contrast to mastectomy) and close followup are the best management option.
